# Characterizing Hepatitis C Virus–Specific CD4^+^ T Cells Following Viral‐Vectored Vaccination, Directly Acting Antivirals, and Spontaneous Viral Cure

**DOI:** 10.1002/hep.31160

**Published:** 2020-11-06

**Authors:** Felicity Hartnell, Ilaria Esposito, Leo Swadling, Anthony Brown, Chansavath Phetsouphanh, Catherine de Lara, Chiara Gentile, Bethany Turner, Lucy Dorrell, Stefania Capone, Antonella Folgori, Eleanor Barnes, Paul Klenerman

**Affiliations:** ^1^ Peter Medawar Building for Pathogen Research University of Oxford Oxford United Kingdom; ^2^ CEINGE–Advanced Biotechnologies Naples Italy; ^3^ Jenner Vaccine Trials Nuffield Department of Medicine University of Oxford Oxford United Kingdom; ^4^ Reithera Srl Rome Italy; ^5^ NIHR Biomedical Research Centre Oxford John Radcliffe Hospital Oxford United Kingdom; ^6^ Translational Gastroenterology Unit John Radcliffe Hospital Oxford United Kingdom

## Abstract

**Background and Aims:**

Induction of functional helper CD4^+^ T cells is the hallmark of a protective immune response against hepatitis C virus (HCV), associated with spontaneous viral clearance. Heterologous prime/boost viral vectored vaccination has demonstrated induction of broad and polyfunctional HCV‐specific CD8^+^ T cells in healthy volunteers; however, much less is known about CD4^+^ T‐cell subsets following vaccination.

**Approach and Results:**

We analyzed HCV‐specific CD4^+^ T‐cell populations using major histocompatibility complex class II tetramers in volunteers undergoing HCV vaccination with recombinant HCV adenoviral/modified vaccinia Ankara viral vectors. Peptide‐specific T‐cell responses were tracked over time, and functional (proliferation and cytokine secretion) and phenotypic (cell surface and intranuclear) markers were assessed using flow cytometry. These were compared to CD4^+^ responses in 10 human leukocyte antigen–matched persons with HCV spontaneous resolution and 21 chronically infected patients treated with directly acting antiviral (DAA) therapy. Vaccination induced tetramer‐positive CD4^+^ T cells that were highest 1‐4 weeks after boosting (mean, 0.06%). Similar frequencies were obtained for those tracked following spontaneous resolution of disease (mean, 0.04%). In addition, the cell‐surface phenotype (CD28, CD127) memory subset markers and intranuclear transcription factors, as well as functional capacity of peptide‐specific CD4^+^ T‐cell responses characterized after vaccination, are comparable to those following spontaneous viral resolution. In contrast, helper responses in chronic infection were infrequently detected and poorly functional and did not consistently recover following HCV cure.

**Conclusions:**

Helper CD4^+^ T‐cell phenotype and function following HCV viral vectored vaccination resembles “protective memory” that is observed following spontaneous clearance of HCV. DAA cure does not promote resurrection of exhausted CD4^+^ T‐cell memory in chronic infection.

**Abbreviations**
aa: amino acids
CCR7: C‐C chemokine receptor type 7
ChAd3: chimpanzee adenovirus
DAA: directly acting antiviral
DMSO: dimethyl sulfoxide
Eomes: Eomesodermin
EOT: end of treatment
FACS: fluorescence‐activated cell sorting
HCV: hepatitis C virus
HLA: human leukocyte antigen
ICS: intracellular cytokine staining
IFN‐γ: interferon‐gamma
IL: interleukin
MHC: major histocompatibility complex
MVA: modified vaccinia Ankara
NS: nonstructural
PBMCs: peripheral blood mononuclear cells
SR: spontaneous viral resolution
T‐bet: T‐box TF TBX21
Tcm: central memory T cells
Tem: effector memory T cells
Temra: effector memory retinoic acid–positive cells
TFs: transcription factors
TNF‐α: tumor necrosis factor alpha
Tscm: stem memory T cells

Hepatitis C virus (HCV) is a global pathogen infecting approximately 71 million people with an estimated 1.75 million new cases annually.^(^ ^1^ ^)^ Following primary infection, most people develop chronic liver disease, which may result in decompensated liver cirrhosis and hepatocellular carcinoma (HCC).^(^ ^2^ ^)^ Despite the introduction of highly efficacious directly acting antiviral (DAA) agents, there remains a strong rationale for preventative HCV vaccination. It is estimated that only 20% of people living with HCV have been diagnosed, largely attributable to the clinically silent nature of the virus, and many present only once liver fibrosis is established.^(^ ^1^ ^)^ Of those diagnosed, only 7.4% of patients have had treatment, in part because of the financial barriers of accessing DAAs.^(^ ^1^ ^)^ Finally, those who are treated remain at risk of reinfection, providing a rationale for a preventative approach.

A T‐cell–mediated vaccine may be an ideal candidate for a preventative HCV strategy. Both CD4^+^ and CD8^+^ T cells have been shown to play a crucial role in immune control against HCV. This was first demonstrated in chimpanzee challenge studies, where it was shown that following previous successful spontaneous viral resolution (SR), antibody‐mediated CD4^+^^(^ ^3^ ^)^ or CD8^+^^(^ ^3^ ^)^ T‐cell depletion led to prolonged viraemia after HCV reinfection. In particular, early robust T‐helper 1 CD4^+^ T‐cell responses are thought to be critical in HCV clearance.^(^ ^4^, ^5^ ^)^ It is hypothesized that functional CD4^+^ T cells prime an effective CD8^+^ T‐cell response against the virus,^(^ ^6^ ^)^ and the absence of this early priming can lead to an exhausted/dysfunctional immune response observed in chronic HCV infection.^(^ ^5^ ^)^

We have previously shown that a heterologous prime‐boost strategy, using chimpanzee adenovirus (ChAd3) and modified vaccinia Ankara (MVA) virus encoding the nonstructural (NS) region of HCV, induces robust HCV‐specific T‐cell responses against a broad range of HCV epitopes in healthy volunteers.^(^ ^7^, ^8^ ^)^ However, these vaccine studies, as well as other studies assessing antigen‐specific T cells in natural HCV infection, have largely focused on the behavior of HCV‐specific CD8^+^ T cells, and there is a paucity of data assessing the behavior of HCV‐specific CD4^+^ T cells following both vaccination and infection. The reasons for this include very small population numbers (typically 0.001%‐0.100%) of CD4^+^ T cells^(^ ^9^ ^)^ and limited tools with which to assess these. Proliferation or stimulation assays are often used; however, these do not allow for accurate assessment of *ex vivo* phenotype and function. In this study, we have used a panel of HCV‐specific major histocompatibility complex (MHC) class II tetramers on large populations of peripheral blood mononuclear cells (PBMCs), in addition to intracellular cytokine staining (ICS), to identify and perform a comprehensive *ex vivo* phenotypic analysis on HCV‐specific CD4^+^ T cells following viral vectored vaccination. We have compared these with the “gold‐standard” CD4^+^ responses observed following SR given that, ideally, vaccination would aim to recapitulate these events. Finally, we have interrogated CD4^+^ T‐cell behavior following DAA treatment given that this group is a target group for vaccination and restoration of CD4^+^ T‐cell function following DAA cure may determine immune response to subsequent vaccination.

## Materials and Methods

### Recruitment of Subjects

#### DAA Patients

Patients with chronic HCV infection (n = 29), including 21 receiving DAA therapy were identified at the John Radcliffe Hospital (Oxford, UK) and recruited following written informed consent. The study protocol conformed to the ethical guidelines of the 1975 Declaration of Helsinki as reflected in a prior approval by the Oxford Biomedical Research Centre (REC 09/H0604/20). Inclusion criteria included a negative HCV viral load at the end of treatment with human leukocyte antigen (HLA) matching the tetramer panel. Additional clinical data were collected (Supporting Fig. S1A,B).

#### Vaccine Trials

Healthy volunteers were recruited at the Churchill Hospital, Oxford into two separate vaccine studies both trialing identical HCV candidate vector vaccines (Endura CT 2009‐018260‐10^(^ ^7^ ^)^ and 2014‐000730‐30^(^ ^10^ ^)^). All volunteers received intramuscular vaccination with experimental vaccines ChAd3‐NSmut1 (ChAd3) and MVA‐NSmut (MVA). Ten volunteers were selected on the basis of a positive enzyme‐linked immunospot response, a matching HLA type for the tetramer panel, and availability of stored vaccine sample (Supporting Fig. S1C,D).

#### Spontaneous HCV Resolution

Individuals with SR were identified from the John Radcliffe Hospital, Oxford defined as HCV‐antibody positive, HCV‐PCR negative. All were treatment naïve, with HLA matching the tetramer panel, and recruited following written informed consent. Where possible, information was gathered about the date of HCV transmission and clearance (Supporting Fig. S1E).

### Vaccines

Development of ChAd3 and MVA vectors have been described.^(^ ^7^, ^11^, ^12^ ^)^ Both vectors encoded the NS3‐5b region of HCV genotype 1b BK strain (1,985 amino acids [aa]). Development of the HCV immunogen has also been described.^(^ ^13^ ^)^

### Peptides, Antigens, and Tetramers

A panel of MHC class II tetramers was donated from the National Institutes of Health Core Tetramer Facility (Atlanta, GA) and Proimmune (Oxford, UK). A total of 11 tetramers were used in the study (Supporting Fig. S2A). Peptides matching the MHC class II tetramer sequence were obtained from Mimotopes. HCV genotype‐1a (H77, Mimotopes Wirral, UK) and genotype‐1b (J4 strain [structural regions] and BK strains [NS regions]) peptides spanning the entire HCV genome were used for vaccinated volunteers and DAA patients (obtained from BEI Resources, Manassas, VA). Each peptide was between 15 and 18 aa in length (overlapping by 11 aa) and arranged in pools representing HCV viral proteins.

### Cell Lines

Short‐term cell lines were used for tetramer and ICS analyses. PBMCs (2‐3 × 10^6^) were stimulated with peptide and costimulatory purified mouse antihuman antibody CD28 (1 µg/mL; BD Biosciences) in 1 mL of RH10 at 37°C. Media were changed and recombinant interleukin (IL)‐2 (50 U/mL; Roche) was added at days 3, 7, and 10. Cells were harvested on day 13 and left to rest overnight in RH10 before ICS and tetramer staining assays.

### MHC Class II Tetramer, Cell‐Surface Marker, and Intranuclear Staining

MHC class II tetramer staining was performed on cultured and *ex vivo* PBMCs. Tetramers were based on immunodominant HCV epitopes described within the NS region.^(^ ^14^, ^15^, ^16^, ^17^ ^)^ Cultured cells were rested overnight before staining, and approximately 2 × 10^5^ PBMCs were used per tetramer. For *ex vivo* tetramer staining, 6‐8 × 10^6^ cells were thawed using RH10 medium with DNase (0.01 mg/mL) before counting (Guava Personal Cell Analysis system). Tetramers were centrifuged for 5 minutes at 14,000*g* at 4°C before staining. Cells were stained with fixable live/dead dye for 5 minutes followed by tetramer staining (phycoerythrin labeled) for 60 minutes at 37°C (1 µg/100 µL) in 50 µL of RH10. Following these, cells were stained with the surface marker panel for 30 minutes. Additionally, for intranuclear staining, cells were then fixed (1% paraformaldehyde), permeabilized (10× perm buffer; eBioscience), and then stained with internal antibody cocktail for 60 minutes (see Supporting Fig. S3 for a full list of antibodies and fluorochromes).

A positive tetramer response was defined as a discrete cluster of cells and >0.004% tetramer^+^/CD4^+^ T cells. This cutoff was determined after an analysis of tetramer^+^ CD4^+^ cells in healthy individuals. In addition, following vaccination, a positive tetramer^+^ CD4^+^ cloud was required to be 3× baseline (prevaccination).

Furthermore, each tetramer was trialed with HLA‐matched and ‐mismatched persons who had not been exposed to either HCV infection or HCV peptides. All tetramers demonstrated clean staining with low background (Supporting Fig. S2B‐G).

### Intracellular Cytokine Stains

ICS was performed following *in vitro* expansion using short‐term cell lines. Before staining, cells were left to rest overnight in 1 mL of RH10 and 37°C. Cells were then plated at 1‐5 × 10^5^ PBMCs/well in 96‐well U‐bottomed plates. PBMCs were stimulated using individual peptides matching the tetramer sequence or pooled peptides F+G+H (matching NS3‐4) plus unstimulated (controlled for dimethyl sulfoxide [DMSO]) and phorbol myristate acetate/ionomycin (50 and 500 ng/mL, respectively). Brefeldin A (10 µg/mL) was added after 1 hour, and cells were stimulated overnight at 37°C. Cells were then stained with fixable live/dead dye, fixed (1% paraformaldehyde), permeabilized (eBiosciences 10× perm buffer), and stained with the antibody panel (Supporting Fig. S3).

All fluorescence‐activated cell sorting (FACS) data were analyzed by an LSRII flow cytometer (BD Biosciences, Franklin Lakes, NJ). Data were collected with BD FACS DIVA software (BD Biosciences, San Jose, CA) and analyzed with TreeStar FlowJo software (FlowJo, LLC, Ashland, OR).

### Statistical Analysis

GraphPad Prism software (version 7; GraphPad Software Inc., La Jolla, CA) was used for all statistical analysis. Nonparametric or parametric two‐tailed tests were used, based on distribution of the population: Paired *t* tests were used for comparisons for matched samples and unpaired *t* tests for unrelated sample comparisons (*P ≤ 0.05; **P ≤ 0.01; ***P ≤ 0.001; ****P ≤ 0.0001). Only statistically significant results were reported in the figures. Unless stated otherwise, all values will be shown as mean population with 95% confidence interval range.

## Results

### HCV Heterologous Prime‐Boost Vaccination With ChAd3 and MVA‐NS Induces HCV‐Specific MHC Class II Tetramer^+^ CD4^+^ T Cells

All vaccinated volunteers chosen for tetramer analysis received priming vaccination with ChAd3‐NSmut1 (dose, 2 × 10^10^ plaque‐forming units), and 8 of 10 volunteers received boosting vaccination with MVA‐NSmut at week 8 (dose, 2 × 10^8^ viral particles [vp]). The remaining 2 volunteers (HCV003347 and HCV003374) received boosting with MVA‐NSmut at week 8 at doses of 2 × 10^7^ and 2 × 10^6^ vp, respectively. Some volunteers also received either an additional round of ChAd3/MVA vaccination or single additional MVA vaccination as part of their trial protocol. Characteristics of vaccine volunteers can be found in Supporting Fig. S1C.

**Fig. 1:**
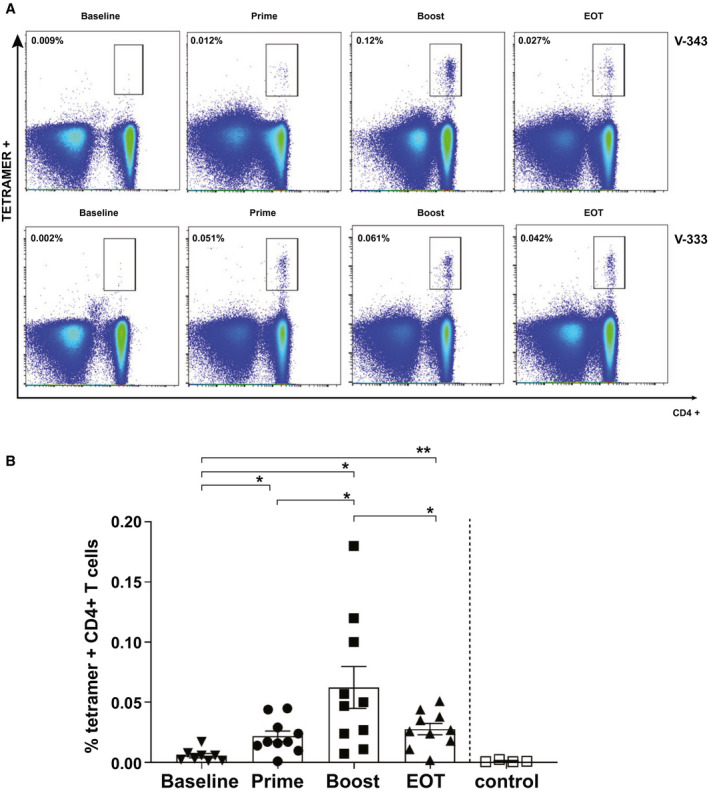
HCV‐specific MHC class II tetramer^+^ CD4^+^ T cells following viral vectored vaccination. (A) Example FACS plot of staining with tetramers 14 HCV‐NS4B_1806‐1818_ in 2 vaccinated healthy volunteers over the study course. Gating is on live CD3^+^ cells. Values indicate the percentage of CD4^+^ T cells binding the tetramer. (B) Percentage of tetramer^+^ CD4^+^ cells after *ex vivo* staining in vaccinated healthy volunteers at different time points of the vaccination regimen. Controls are represented by healthy volunteers stained with mismatched MHC class II tetramer. Error bars represent the SEM.

In these volunteers, MHC class II tetramer‐positive populations were assessed over time; at baseline before vaccination, after prime vaccination, after boost vaccination, and at the end of study. Examples plots are shown in Fig. 1A for 2 vaccinated volunteers at different time points.

The highest frequency of HCV‐specific T cells was observed following boosting vaccination with MVA (mean, 0.062% [0.023‐0.10] of CD4^+^ T cells). The population was significantly larger compared with other trial time points. The control population, healthy HLA‐mismatched PBMCs not exposed to HCV virus or vaccination, was comparable to baseline staining (Fig. 1B).

### Robust and Durable *Ex Vivo* Tetramer‐Positive CD4^+^ T‐Cell Populations are Detectable also in SR, But Not in Chronic HCV, Patients Following DAA Therapy

We then sought to compare the HCV tetramer‐specific CD4^+^ T‐cell population induced following vaccination to a natural model of HCV clearance (SR) as well as to cured HCV infection with DAA therapy. Given the widely published data suggesting negligible or absent HCV‐specific CD4^+^ T cells in chronic infection,^(^ ^15^, ^18^ ^)^ we were interested whether these populations recover following DAA‐mediated viral clearance.

Ten samples from volunteers with SR and 21 patients receiving DAA therapy were selected (where HLA matched available tetramers). Samples were taken between 0 and 52 weeks before commencing treatment (pre‐DAA) and, on average, 6 weeks following treatment completion (range, 0‐26 weeks; post‐DAA).

**Fig. 2:**
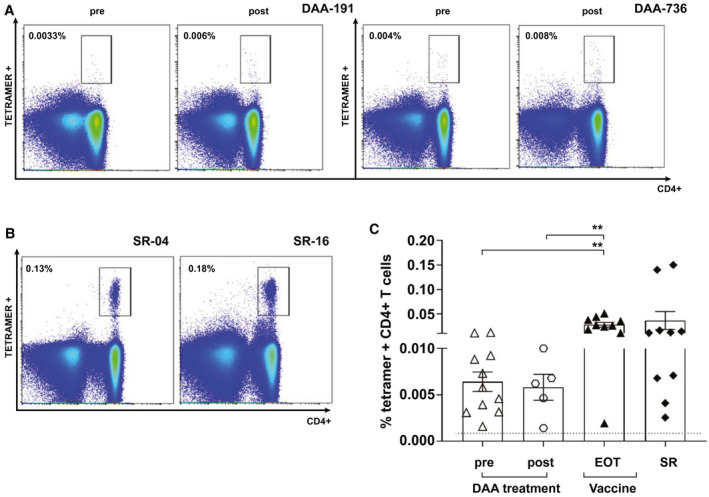
Assessment of *ex vivo* tetramer^+^ CD4^+^ T cells pre‐DAA and post‐DAA treatment, following vaccination and in SR volunteers. (A,B) FACS example plot of staining with tetramers 14 HCV‐NS4B_1806‐1818_, 24 HCV‐NS3_1535‐1551_ or pool of tetramers 17 HCV‐NS3_1582‐1597_, tetramer 18 HCV‐NS3_1411‐1425_, and tetramer 19 HCV‐NS3_1535‐1551_ in 2 DAA patients pretherapy and posttherapy and in 2 SRs. Gating is on live CD3^+^ cells. Values indicate the percentage of CD4^+^ T cells binding the tetramer. (C) Percentage of tetramer^+^ CD4^+^ cells after *ex vivo* staining at the end of study of vaccinated healthy volunteers, DAA pretherapy and posttherapy, and in SR persons. Error bars represent the SEM. Only statistical differences are shown.

MHC class II tetramer^+^ CD4^+^ T cells were detectable following SR (mean, 0.036% [0.0‐0.08] of total CD4^+^ T cells; Fig. 2B,C). This population was comparable in magnitude to the vaccine groups, in particular at the final trial time point (mean, 0.027% [0.017‐0.038]). There were few detectable CD4^+^ tetramer‐positive T cells following *ex vivo* staining in the DAA‐treated patient groups at either time point (Fig. 2A,C).

### HCV Vaccination Induces High Levels of Long‐Lived Memory (CD127) and Costimulatory (CD28) Markers in *Ex Vivo* HCV‐Specific CD4^+^ T Cells Analogous to Spontaneous Resolution

We assessed the phenotypic profile of *ex vivo* HCV‐specific tetramer^+^ CD4^+^ T cells in chronic HCV patients, vaccinated volunteers, and SR persons. Following staining with MHC class II tetramers, PBMCs were stained with a surface panel containing costimulatory and memory markers (gating strategy showed in Supporting Fig. S4).

**Fig. 3:**
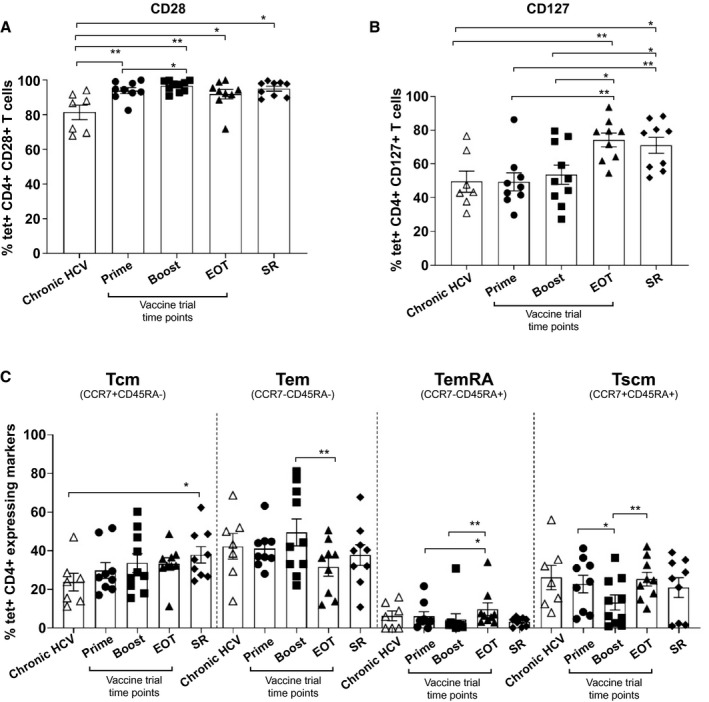
Analysis of costimulatory and memory cell‐surface markers in chronic HCV, vaccinated, and SR volunteers. (A,B) Thawed PBMCs were costained with MHC class II tetramers and anti‐CD127 and ‐CD28 antibodies in chronic HCV patients, vaccinated volunteers at different trial time points, and in SR persons. (C) Proportion of tetramer^+^ cells expressing Tcm, Tem, Temra, and Tscm phenotype in the same cohorts. All tetramer staining and phenotyping were performed *ex vivo*. Error bars represent the SEM. Only statistical differences are shown.

CD28 (a critical costimulatory molecule for T‐cell activation) was highly expressed at all time points after vaccination, with peak expression postboost (mean, 96.6% [94.3‐98.9]). CD28 expression was significantly lower in patients with chronic HCV (mean, 81.4% [71.1‐91.8]; Fig. 3A).

CD127 (IL7R) expression of tetramer^+^ T cells increased during the course of the trial and reached peak expression at end of treatment (EOT) time point (mean, 74.3% [64.8‐83.8]). CD127 expression at EOT was comparable to that observed in SR (mean, 71.05% [60.02‐82.1]), and both these groups demonstrated significantly higher expression compared to both prime and boost vaccination and chronic HCV patients (Fig. 3B).

Memory subsets were further assessed as follows: stem memory T cells (Tscm; CCR7^+^/CD45RA^+^), effector memory T cells (Tem; CCR7^−^/CD45RA^−^); central memory T cells (Tcm; CCR7^+^/CD45RA^−^); and effector memory retinoic acid–positive cells (Temra; CCR7^−^/CD45RA^+^).

Central and effector memory subsets were the most predominant at all vaccine time points as well as in SR (Fig. 3C). The percentage of Tcm cells was similar between these groups, ranging from 29.9% (20.3‐39.1) to 38% (28.15‐47.7) of tetramer^+^ CD4^+^ T cells (prime and SR, respectively). Chronic HCV patients comparatively had lower expression (mean, 23.8% [12.5‐35]), significantly so when compared to SR (*P* = 0.04).

Similarly, robust Tem populations were observed in all groups and was highest following boosting vaccination (49.6% [33.8‐65.6]), reducing significantly by EOT (mean, 31.6% [20.9‐42.4]; (*P* = 0.0075). Populations of Tscms were observed to contract between prime and boost vaccination (mean, 22.8% [12.4‐33.3] to 13.2% [4.5‐21.8]; *P* = 0.01%) and then re‐expand at the end of the trial (mean, 25.3% [17.2‐33.5]; *P* = 0.005%).

Temras were the smallest population observed out of the memory marker subsets. However, numbers increased significantly at the end of the trial (mean, 9.8% [2.1‐17.5]) compared with prime (mean, 6.1% [0.75‐11.5]; *P* = 0.01) and boost vaccination (mean, 4.6 [0.0‐11.2]; *P* = 0.007; Fig. 3C).

### T‐box TF TBX21 and Eomesodermin Expression Following Vaccination Mirrors SR

**Fig. 4:**
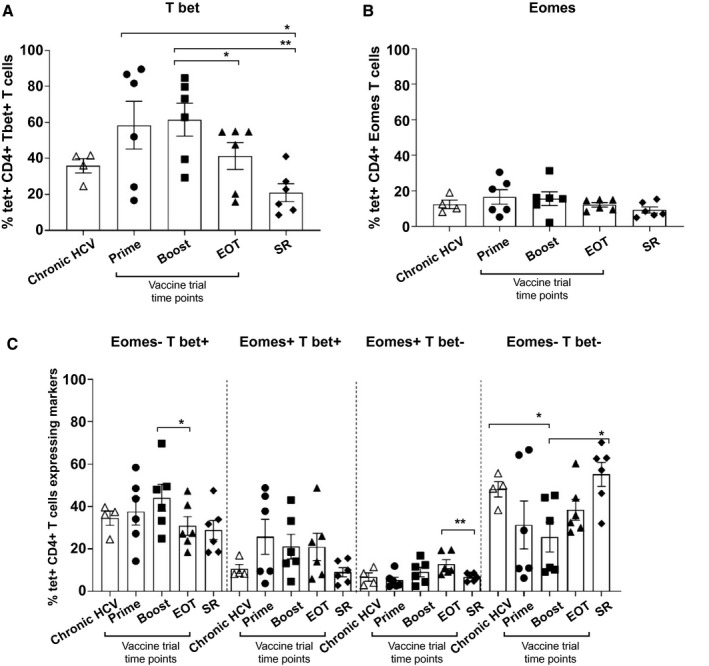
TF analysis in chronic HCV, vaccinated, and SR volunteers. (A,B) Percentage of tetramer^+^ CD4^+^ T cells expressing T‐bet and Eomes in chronic HCV patients, vaccinated volunteers, and SR persons. (C) Costaining with class II tetramers and anti‐human T‐bet and Eomes antibodies in the same cohorts. Error bars represent the SEM. Only statistical differences are shown.

Intranuclear phenotypic analysis was performed on two transcription factors (TFs) important in T‐cell activation and differentiation into effector and memory cells: T‐box TF TBX21 (T‐bet) and Eomesodermin (Eomes). Robust T‐bet expression was demonstrated following prime and boost vaccination, but, however, was reduced at the EOT (mean, 41.4% [15.9‐55] of tetramer^+^ CD4^+^ T cells), which reached significance when compared with T‐bet staining following boosting vaccination (mean, 61.5% [29.3‐84.6]; *P* = 0.03). T‐bet expression in SR was lower than all other groups, reaching significance when compared with prime (*P* = 0.04) and boost vaccination (*P* = 0.009; Fig. 4A). There are fewer published studies assessing Eomes behavior in CD4^+^ T cells.^(^ ^19^, ^20^ ^)^ Expression of Eomes was low at all vaccine time points as well as in chronic HCV patients and in SR individuals (Fig. 4B).

Furthermore, there is a growing body of evidence that changing levels of T‐bet and Eomes expression occur on the pathway to T‐cell exhaustion resulting in a final T‐bet^lo^/Eomes^hi^ population.^(^ ^21^ ^)^ This exhausted population was very low in chronic HCV patients, and in each time point after the study, although it was significantly higher at the EOT compared to SR (*P* = 0.0087; Fig. 4C).

In summary, expression of T‐bet significantly decreased over the course of the trial and was lowest in SR.

### Proliferative Capacity of HCV Tetramer‐Specific CD4^+^ T Cells Following HCV Vaccination Is Robust, But Limited Following DAA Mediated Cure

**Fig. 5:**
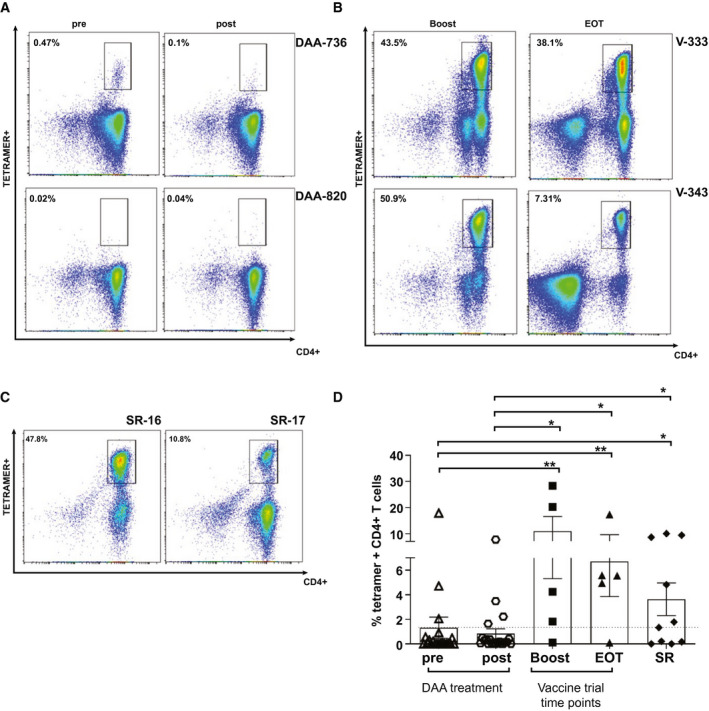
Proliferative capacity of CD4^+^ T cells in pre‐DAA– and post‐DAA–treated patients, vaccinated, and SR volunteers. (A‐C) Example FACS plots of tetramer^+^ CD4^+^ T cells after 14 days of culture with peptide matching tetramer sequence in 2 DAA patients pretreatment and posttreatment (A), 2 vaccinated volunteers at boost and EOT (B), and 2 SR persons (C). Values indicate the percentage of CD4^+^ T cells binding tetramer. (D) Scatter plot with bar showing the percentage of tetramer^+^ CD4^+^ T cells after culture in pre‐DAA and post‐DAA, vaccination, and SR. Error bars represent the SEM. Only statistical differences are shown.

We sought to assess the proliferative capacity of HCV‐specific CD4^+^ T cells following DAA‐mediated cure (n = 21) and compare with HCV vaccination (n = 5) and SR (n = 10). Proliferative capacity of HCV‐specific CD4^+^ T cells in all groups was assessed using MHC class II tetramers following *in vitro* culture with peptide corresponding to the tetramer and IL‐2 for 2 weeks (Fig. 5A‐D). Between one and six tetramers were used with each sample (depending on HLA specificity), with the highest‐magnitude tetramer for each individual sample chosen for analysis (Supporting Fig. S1D).

There was a robust population of HCV tetramer^+^ CD4^+^ T cells induced following viral vectored vaccination (mean, 10.94% [0.0‐26.6] at boost and 6.7% [0.0‐14.6] at EOT). Likewise, there was a comparable, though smaller, population in SR (mean, 3.6% [0.6‐6.6]; Fig. 5B‐D). In comparison, there was a poor proliferative response in the DAA patient cohorts (mean, 1.3% [0.0‐3.1] and 0.8% [0.02‐1.70] for pre‐DAA and post‐DAA, respectively; Fig. 5A,D). There was no clear pattern or trend between the pre‐DAA and post‐DAA treatment groups, with subgroup analyses comparing patients who had both a 2‐fold increase and 2‐fold decrease in tetramer^+^ populations showing no significant differences in phenotypic characteristics (Supporting Fig. S5).

Single‐epitope sequences that were contained within the tetramers were compared following *in vitro* culture to assess the immunogenicity of each. The peptide sequence contained in tetramer 14 (NS3_1806‐1818_) restricted to HLA DRB1*0101 was, by far, the most immunogenic across all four groups. In comparison, T‐cell populations binding tetramers 22 (NS2_794‐810_) and 29 (NS5A_1957‐1975_) were rarely detectable (Supporting Fig. S6).

### Functionality of CD4^+^ T Cells in Pre‐DAA and Post‐DAA Treatment, Vaccinated, and SR Groups

**Fig. 6:**
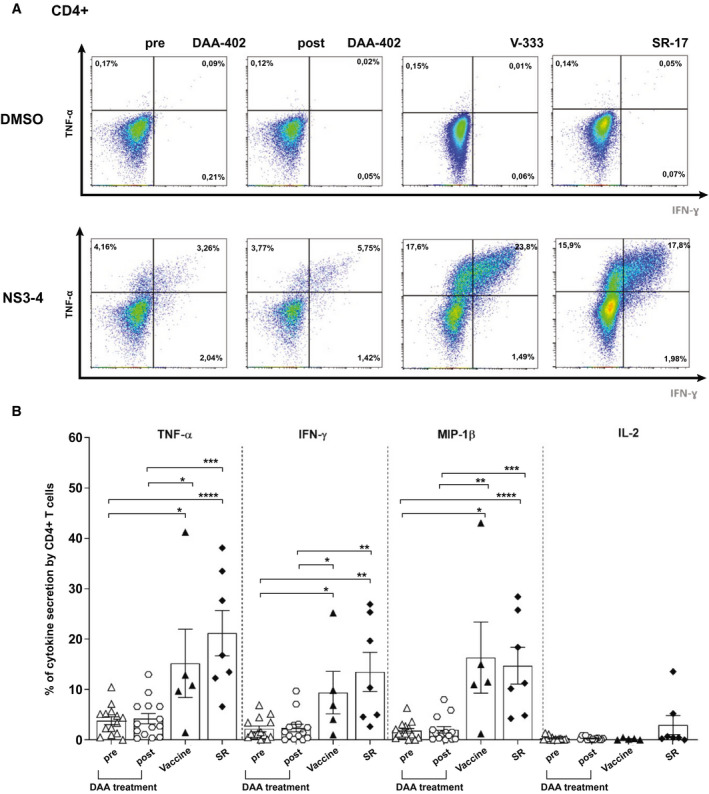
Functional capacity of CD4^+^ T cells in pre‐DAA– and post‐DAA–treated patients, vaccinated, and SR volunteers. (A) Example FACS plots showing TNFα/IFN‐γ after intracellular cytokine staining are shown for CD4^+^ T cells stimulated with NS3‐4 or DMSO control in DAA patients pretreatment and posttreatment, vaccine volunteers (after boost vaccination), and SR. (B) Comparison of cytokine production by CD4^+^ T cells pre‐DAA (n = 14) and post‐DAA treatment (n = 14), after ChAd3/MVA vaccination (n = 5), and in SR (n = 7). PBMCs were cultured for 14 days with peptide matching NS3‐4 (pools F+G+H), rested, and restimulated with the same peptides overnight. Staining in DMSO wells was subtracted. Error bars represent the SEM. Only statistical differences are shown.

To evaluate the capacity of cytokine secretion following HCV vaccination, SR, and following DAA treatment, ICS assays were performed following *in vitro* expansion using peptide pools F‐H (corresponding to NS3‐4), CD28, and periodic addition of IL‐2. These peptide pools were chosen based on their immunogenicity demonstrated in HCV vaccine trials to date^(^ ^7^, ^8^ ^)^ as well as published data identifying a number of immunogenic CD4^+^ epitopes in NS3‐4 proteins.^(^ ^22^, ^23^ ^)^ Representative ICS plots are shown in Fig. 6A.

Overall, there was poor cytokine production in the DAA patient cohort, with no recovery of cytokine production observed following viral cure and no significant differences between the two groups (Fig. 6B). In comparison, vigorous cytokine production was observed for three of the four cytokines tested in both SR and following vaccination. Production of tumor necrosis factor alpha (TNF‐α), interferon‐gamma (IFN‐γ), and macrophage inflammatory protein 1 beta (MIP‐1β) was significantly greater in these groups compared to both pre‐DAA and post‐DAA treatment, peaking at 21.2% (10.18‐32.20) for TNF‐α production in SR. No significant difference was observed between SR and vaccination in any measured cytokine (Fig. 6B). We observed a strong correlation of cytokine production between CD4^+^ and CD8^+^ T cells measured at the same time points after vaccination (*r* = 0.73; *P* ≤ 0.0001; Supporting Fig. S7).

## Discussion

This research addresses two important areas of HCV research; the behavior of viral‐specific CD4^+^ T cells following HCV vaccination and the extent of immune recovery following DAA‐mediated HCV cure. CD4^+^ T cells are widely believed to play a vital role in the early immune response to HCV infection. However, HCV‐specific CD4^+^ T cells have been challenging to study, largely because of limited/absent populations and limited tools for assessing them *ex vivo*. The use of MHC class II tetramers to predictably assess CD4^+^ responses has been limited by the promiscuous epitope binding to MHC class II alleles^(^ ^17^ ^)^ and a variable length of amino acids optimal for binding to the MHC.^(^ ^24^ ^)^ Here, we used a panel MHC class II tetramers, ICS, and peptide‐stimulated cell lines to perform a detailed analysis of the functional and phenotypic behavior of CD4^+^ T cells following HCV viral vectored vaccination in chronic HCV infection before and following DAA‐mediated HCV cure and in SR.

Using large input cell numbers (6‐8 × 10^6^ cells), we were able to detect HCV‐specific tetramer^+^ CD4^+^ T cells following ChAd3/MVA prime‐boost viral vectored vaccination in *ex vivo* analysis. Tetramer populations were identified at every time point after vaccination and peaked following boosting vaccination, comparable to the kinetics of CD8^+^ T cells following identical vaccination schedules.^(^ ^7^ ^)^ Within our panel of 10 tetramers, we observed variable peptide sequence immunogenicity; however, epitopes restricted to NS3, notably DRB1*01 restricted sequence NS3_1806‐1818_, were the most immunogenic, supporting previous publications.^(^ ^15^ ^)^ Importantly, the size of the tetramer^+^ cell population at the final trial time point, and thus the best predictor of the residual memory population following vaccination, was comparable to that observed in SR.

Although the number of HCV‐specific CD4^+^ T cells induced by vaccination is undoubtedly important, the phenotypic and functional properties of CD4^+^ T cells are likely to be significant determinants of a protective response. We hypothesize that vaccine‐induced CD4^+^ T cells that function in an analogous way to CD4^+^ T cells following SR may be predictive of a protective response. We analyzed a number of cell‐surface markers associated with T‐cell activation (CD28) and memory differentiation (CD127, C‐C chemokine receptor type 7 [CCR7], and CD45RA) in chronic HCV patients, vaccinated volunteers, and SR individuals, using tetramer^+^ populations in *ex vivo* CD4^+^ T cells.

A critical component of a successful vaccine is one that induces long‐lived memory cells capable of homeostatic proliferation,^(^ ^25^ ^)^ with appropriate costimulatory molecules. CD28 is a critical costimulatory molecule in T‐cell activation,^(^ ^26^ ^)^ and CD4^+^CD28^–^ T cells are widely observed to be present in chronic viral infections, such as cytomegalovirus,^(^ ^27^ ^)^ human immunodeficiency virus,^(^ ^28^ ^)^ and hepatitis B virus.^(^ ^29^ ^)^ CD28 was highly expressed at all time points after vaccination. Significantly lower levels were expressed in chronic HCV patients compared to vaccinated and SR volunteers. CD127 (IL‐7rα) is strongly associated with T‐memory‐cell development. Using adoptive transfer models in mice, Kaech et al. showed that expression of CD127 was required as a precursor for functional memory‐cell development.^(^ ^30^ ^)^ IL7 (the ligand for CD127) has been shown to play a critical role in maintenance of a polyclonal and functionally diverse repertoire of human CD4(+) memory T cells in the absence of ongoing antigen stimulation.^(^ ^31^ ^)^ We have shown CD127 expression to be progressively up‐regulated in *ex vivo* HCV tetramer^+^ CD4^+^ T cells following HCV vaccination, suggesting that this marker is associated with memory‐cell development. When compared to expression in natural HCV clearance, expression was similar to the final trial time point (mean, 74.3% at EOT and 71% for the SR group) and significantly lower in chronic HCV patients (mean 49%).

Much work has been directed at elucidating and characterizing T‐cell memory subsets with a focus on CD8^+^ T cells and a paucity of data assessing CD4^+^ T‐memory subsets. Here, we provide a valuable insight into the behavior of these cell populations in tetramer^+^ CD4^+^ T cells in chronic HCV patients, vaccinated volunteers, and SR individuals. In all settings, Tcms and Tems were the predominant memory subset induced, in keeping with published data in CD8^+^ populations. These two populations are perhaps the most widely studied, and both contribute critical functions to a successful memory response—rapid differentiation on antigen re‐exposure (Tems) and long half‐life and proliferative capacity (Tcms). Tscms have been recently described as long‐lived multipotent memory cells.^(^ ^32^, ^33^ ^)^ We show a robust population of Tscms in all study groups. Notably, Tscm expression expanded significantly between boosting and EOT (mean, 13.2%‐25.3%), similar to previous observations in CD8^+^ T cells following ChAd3/MVA prime boost.^(^ ^7^ ^)^ CD45RA re‐expression in long‐lived memory cells is important for the ability to self‐renew, and to differentiate into other memory subsets, whereas CCR7 re‐expression is important for exposure to circulating antigen—both critical components of memory cells. They have been suggested to play a role in the persistence of latent HIV reservoirs in CD4^+^ T cells of infected hosts,^(^ ^34^ ^)^ but their role in CD4^+^ T cells in maintaining protection following both viral clearance and vaccination—although presumed—has been challenging to identify. We observed small populations of Temras in all groups, consistent with previous observations in CD4^+^ T cells,^(^ ^35^ ^)^ and, similarly to Tscm, the expansion of this subset between boosting and EOT may reflect an evolving CD4^+^ memory phenotype with time. The role of Temras in CD4^+^ T cells has not been fully elucidated, although they have been associated with CX3C chemokine receptor 1 expression, suggesting a potential cytotoxic phenotype.^(^ ^36^, ^37^ ^)^

TF analysis was performed on tetramer‐gated *ex vivo* CD4^+^ T cells specific for HCV. T‐bet, a central regular in CD4^+^ Th1 differentiation and widely studied in both CD4^+^ T and CD8^+^ subsets, was observed to decrease in expression over time following vaccination, reflecting previous observations that T‐bet expression declines as CD4^+^ T cells gain a more memory‐like phenotype.^(^ ^20^ ^)^ The reduced T‐bet expression in SR (on average, sampled many years post‐HCV exposure) further supports this. Unlike its CD8^+^ counterpart, there is a lack of literature assessing Eomes expression in CD4^+^ T cells following antigen exposure. Knowledge to date is largely confined to assessing Eomes in bulk CD4^+^ phenotyping, with low expression observed, particularly in Tcms compared with Tems and effector cells.^(^ ^19^, ^20^ ^)^ We observed low levels of Eomes expression throughout the vaccine trial and in SR, in keeping with published data. Importantly, the comparable expression of both TFs following HCV vaccination—particularly at the final trial time point—with SR indicates that vaccination may induce a memory CD4^+^ T cell with a protective transcriptional signature.

In the chronic HCV group, we observed the memory phenotype and TF expression of HCV tetramer^+^ CD4^+^ T cells to be similar to all vaccine time points and SR, with the exception of a significant decrease in Tcm in chronic HCV compared to SR. A possible explanation for this similarity is selection bias, in that those CD4^+^ T cells detectable in chronic infection are relatively functionally preserved, while the most functionally exhausted are deleted.

The ability of antigen‐specific memory T cells to rapidly expand and produce cytokine upon re‐exposure to antigen is a critical component of a functional and protective cell‐mediated response.^(^ ^38^, ^39^, ^40^ ^)^ Following peptide‐stimulated short‐term culture, we observed robust proliferation as well as effector cytokine production after ChAd3/MVA vaccination, comparable to SR. In particular, antiviral cytokines IFN‐γ and TNF‐α were readily produced following ICS stimulation, which are well‐defined correlates of highly effective T cells. IL‐2 production was attenuated; progressively diminishing IL‐2 production has previously been observed following T‐cell activation through a negative feedback loop.^(^ ^41^, ^42^ ^)^ We hypothesize that poor IL‐2 secretion was attributed to IL‐2 down‐regulation following activation and proliferation in cell lines. Overall, these results mirror the behavior of HCV‐specific CD8^+^ T cells following vaccination,^(^ ^7^, ^8^ ^)^ and there was a high correlation of cytokine production between CD4^+^ and CD8^+^ T cells, suggesting a coordinated response to vaccination. Similar phenotypic and functional correlations between peptide‐specific CD4^+^ and CD8^+^ T cells have been observed in highly effective viral vaccines, such as yellow fever vaccine.^(^ ^43^ ^)^ The fact that CD4^+^ vaccine‐induced responses are phenotypically and functionally analogous to that observed in natural HCV clearance is suggestive of a protective immune response. Importantly, our observations in SR, made on average many years or decades following exposure, suggests that the phenotype observed is long‐lived, as suggested by previous studies.^(^ ^44^ ^)^

The other critical question addressed here is the state of the host immune system after DAA‐mediated HCV cure. T‐cell exhaustion, or the hierarchical loss of effector functions and eventual anergy and deletion,^(^ ^45^ ^)^ is a well‐described process in chronic infection, including HCV.^(^ ^46^, ^47^ ^)^ The new era of DAA treatment gives a unique opportunity to interrogate immunological recovery following cure of chronic viral infection. Using tetramer assays and ICS following peptide stimulation, we were unable to detect an increase in proliferative capacity or production of antiviral cytokines following DAA‐mediated HCV cure. There are a number of hypotheses to explain this. First, more time may be needed for the host immune system to “reset” following cure and functional ability to be restored. Second, antigen repriming with naïve thymic emigrants may be required following viral cure to induce a functional immune response. Third, all the patients in this cohort had liver cirrhosis, well described to induce an immunosuppressive state in the host.^(^ ^48^ ^)^ A final hypothesis, however, is that the host HCV‐specific immune response in chronic HCV infection is terminally exhausted and unable to be reversed following cure. Our observations contrast with a previous study demonstrating T‐cell recovery following IFN‐free therapy^(^ ^49^ ^)^; the study by Martin et al. assessed CD8 T cells in treatment‐naïve patients without cirrhosis, suggesting that the limited recovery observed in our population may be attributable to previous or current IFN treatment or presence of cirrhosis. However, HCV reinfection is known to occur following DAA treatment in those with ongoing HCV exposure, including patients without cirrhosis. These clinical data show that DAAs do not consistently restore HCV‐specific immune responses to protect against reinfection. An important remaining question is whether HCV‐specific immune responses can be restored following DAA cure to a level that will enable effective HCV‐specific vaccination strategies. Clinical trials in progress (NCT03688061) will address this question directly.

In summary, we provide a detailed analysis of phenotypic and functional characteristics of HCV‐specific CD4^+^ T cells. Following vector‐based HCV vaccination, HCV‐specific CD4^+^ T cells are analogous to those in the gold‐standard setting of SR in both phenotypic and functional parameters, suggesting the induction of effective and protective CD4^+^ T cells following vaccination. Furthermore, we show minimal functional recovery of the same population of cells following DAA‐mediated cure. This work has wider implications to both T‐cell vaccine development and chronic viral infection. Further studies assessing both *in vivo* responses to HCV vaccines and behavior of CD4^+^ T cells following vaccination in DAA‐mediated cure are required.

## Author Contributions

E.B., F.H., L.D., S.C., A.F. and P.K. contributed to conception and design. F.H., I.E., L.S., A.B., C.P., C.L., C.G., B.T., J.K. contributed to acquisition analysis. E.B., F.H., I.E. and P.K. contributed to the interpretation of the data. E.B., F.H. and I.E. drafted the article, revised critically for important intellectual content and worked for a final approval of version to be published.

## Supporting information

Supinfo
