# Supplementary material for: Characterizing Hepatitis C Virus–Specific CD4+ T Cells Following Viral‐Vectored Vaccination, Directly Acting Antivirals, and Spontaneous Viral Cure
Source: Hepatology. 2020 Nov 6;72(5):1541–55. doi: 10.1002/hep.31160 (PMC7610807; doi:10.1002/hep.31160)
Supplement: Supplementary file 1 — Supinfo [file HEP-72-1541-s001.pdf]

Supplementary Figures

A Patients included in the pre and post DAA analysis

N	Geno	Cirrhosis present	Previous Treatment	Current Treatment	HCV PCR negative	1st Timepoint	2nd timepoint	HLADR	Best association tetramer/allele
**261**	1b	Y	IFN, PI	S+L+riba	TW4	3mth pre	3mth post	DRB1*01,03	14
**383**	1	Y	IFN	S+L+riba	TW8	11mth pre	1mth post	DRB1*07	24
**402**	1	Y	IFNx2, PI	S+L+riba	TW6	7mth pre	3mth post	DRB1*01	14
**890**	1	Y	IFN	S+L+riba	TW4	6mth pre	1mth post	DRB1*03,07	24
**895**	1b	Y	IFNx2, PI	S+L+riba	TW8	1mth pre	3mth post	DRB1*15	17,18,19
**913**	1a	Y	IFN	S+L+riba	TW8	6mth pre	3mth post	DRB1*04,15	17,18,19
**996**	1	Y	IFNx2, PI	S+L+riba	TW4	12mth pre	1mth post	DRB1*04	15,20,21
**1119**	1a	Y	Nil	S+L+riba	TW4	12mth pre	1mth post	DRB1*01	14
**191**	1	Y	IFNx2, PI	S+L+riba	TW4	4mth pre	6mth post	DRB1*07	24
**185**	3	N	Nil	S+L+riba	TW8	48mth pre	0mth post(EOT)	DRB1*07,15	17,18,19
**202**	1b	N	IFN	O+P+R+D	TW8	0mth pre(SOT)	0mth post(EOT)	DRB1*07	24
**722**	3	Y	IFN	S+V	TW8	0mth pre(SOT)	0mth post(EOT)	DRB1*01,15	14
**736**	3	N	IFN	S+IFN+riba	TW8	6mth pre	3mth post	DRB1*03,15	17,18,19
**820**	1b	N	IFN	S+L+riba	TW8	0mth pre(SOT)	0mth post(EOT)	DRB1*04	15,20,21
**950**	1a	N	Nil	S+L+riba	TW8	0mth pre(SOT)	1mth post	DRB1*04	15,20,21
**984**	1a	N	IFN	S+L+riba	TW4	0mth pre(SOT)	0mth post(EOT)	DRB1*01	14
**1164**	1	Y(T)	Nil	S+L+riba	TW4	6mth pre	0mth post(EOT)	DRB1*03,04	15,20,21
**1215**	3a	YN	IFN	S+IFN+riba	TW4	6mth pre	3mth post	DRB1*04	15,20,21
**238**	3	N	IFN	S+IFN+riba	TW4	1mth pre	0mth post(EOT)	DRB1*03,07	24
**780**	1	Y	IFN, PI	S+L+riba	TW8	6mth pre	3mth post	DRB1*15	17,18,19
**1087**	3	N	IFNx2	S+V	TW2	12 mth pre	3 mth post	DRB1*07	24

B Chronic HCV patients included in the phenotypic analysis (Surface and TF markers)

N	Geno	Cirrhosis present	HLADR	Best association tetramer/allele
**1065**	1b	Y	DRB1*01,15	14
**876**	1a	Y	DRB1*07	24
**1262**	1b	Y(T)	DRB1*01	14
**638**	1a	Y	DRB1*04,07	24
**1247**	1a	Y	DRB1*07	24
**648**	1	na	DRB1*15	17,18,19
**1254**	1a	Y(T)	DRB1*15	17,18,19
**127**	1	N	DRB1*04,07	24
**191**	1	Y	DRB1*07	24
**402°**	1	Y	DRB1*01	14
**736°**	3	N	DRB1*03,15	17,18,19
**890°**	1	Y	DRB1*03,07	24
**1164°**	1	Y(T)	DRB1*03,04	15,20,21
**261°**	1b	Y	DRB1*01,03	14
**820°**	1b	N	DRB1*04	15,20,21
**913°**	1a	Y	DRB1*04,15	17,18,19
**950°**	1a	N	DRB1*04	15,20,21
**996°**	1	Y	DRB1*04	15,20,21
**202°**	1b	N	DRB1*07	24
**984°**	1a	N	DRB1*01	14

C Vaccine volunteers (*ex-vivo*)

Trial number	Prime	Boost	EOT	HLA-DR	Tetramer
HCV003333^^	TW2	TW9	TW32	DRB1*01	14
HCV003335^^	TW2	TW12	TW32	DRB1*04, *15	15, 17, 18, 19, 20, 21
HCV003343^	TW2	TW12	TW34	DRB1*01	14
HCV003345^	TW2	TW9	TW34	DRB1*04, *15	15, 17, 18, 19, 20, 21
HCV003347**	TW4	TW12	TW34	DRB1*15	17, 18, 19
HCV003374***	TW4	TW9	TW34	DRB1*01	14
PEA-04009	TW2	TW9	TW34	DRB1*01	14
PEA-04011	TW4	TW12	TW34	DRB1*15	17, 18, 19
PEA-04014	TW2	TW9	TW34	DRB1*15	17, 18, 19
PEA-04028	TW2	TW9	TW34	DRB1*15	17, 18, 19

D Vaccine volunteers (*in vitro*)

Trial number	Time point	Vaccination schedule	HLA-DR	Tetramer
HCV003333^	TW9	AdCh3 (TW0) / MVA (TW8)	DRB1*01	14
HCV003335^	TW12	AdCh3 (TW0) / MVA (TW8)	DRB1*04, *15	15, 17, 18, 19, 20, 21
HCV003337^	TW32	AdCh3 (TW0) / MVA (TW8)	DRB1*04	15, 20, 21
HCV003339	TW48	AdCh3 (TW0) / MVA (TW8) / MVA (TW40)	DRB1*07	22, 24, 29
HCV003343	TW18	AdCh3 (TW0) / MVA (TW8) / MVA (TW40)	DRB1*01	14

E Spontaneous resolvers individuals

Cohort number	Year of HCV Transmission	HLA-DR	Tetramer
SR3	Unknown	DRB1*01, *04	14, 15, 20, 21
SR4	1980's	DRB1*03, *15	17, 18, 19
SR8	Unknown	DRB1*07	22, 27, 29
SR9	1970's	DRB1*07	22, 27, 29
SR10	1980's	DRB1*15	17, 18, 19
SR11	Unknown	DRB1*04	15, 20, 21
SR13	Unknown	DRB1*15	17, 18, 19
SR14	2000's	DRB1*07, *15	17, 18, 19, 22, 27, 29
SR16	1990's	DRB1*01, *07	14, 22, 27, 29
SR17	1990's	DRB1*15	17, 18, 19

Supplementary Figure 1: List of participants in three analysed cohorts.
A) Characteristics of patients included in pre and post DAA analysis. N=cohort number, Geno=genotype, Y=yes, N=no, T0=liver transplant, IFN= interferon therapy, Riba=oral ribavirin, PI= protease inhibitor (telaprevir, boceprevir), S=sofosbuvir, L=ledipasvir, V=velpatisvir, O=ombitasvir, P=paritaprevir, R=ritonavir, D=dasabuvir. TW=treatment week, EOT= end of treatment, SOT=start of treatment.
B)Chronic HCV patients included in the phenotypic analysis. ° indicates that some patients included in the table B subsequentially received a DAA treatment and are also included in table A, N/A= not available.
C) Vaccine trial volunteers used in ex-vivo tetramer analysis. Healthy volunteers vaccinated with AdCh3 NSmut1 2.5 x10^10 vp (prime vaccine) and MVA-NSmut 2x10^8 pfu, 2x10^7 pfu (**) or 2x10^6 pfu (***) (boost vaccine). ^volunteers received an additional MVA at week 40. ^^volunteers received an additional round of AdCh3/MVA 21 months earlier

(HCV003333) and 11 months earlier (HCV003335). Note 'baseline' in these samples was prior to first vaccination round.
D) Vaccine trial volunteers used in in-vitro cell line analysis. Healthy volunteers vaccinated with AdCh3/MVA as per above schedule. ^volunteers received previous vaccination with AdCh3/MVA 21 months prior (HCV003333) and 11 months prior (HCV003335, HCV003337).
E) Characteristics of individuals with spontaneous HCV clearance. Volunteers recruited based on being HCV antibody positive, HCV PCR negative, the absence of previous HCV treatment and HLA matching to the MHC Class II tetramer panel

A

N	Protein	Position	Peptide Sequence	HLA restriction
**14**	NS4b	1806-1818	TLLFNILGGWVAA	DRB1*0101
**15**	NS3	1248-1262	GYKVLVLNPSVAATL	DRB1*0401
**17**	NS3	1582-1597	NFPYLVAYQATVCARA	DRB1*1501
**18**	NS3	1411-1425	GINAVAYYRGLDVSV	DRB1*1501
**19**	NS3	1535-1551	TTVRLRAYMNTPGLPVC	DRB1*1501
**20**	NS4b	1771-1790	SGIQYLAGLSTLPGNPAIASL	DRB1*0401
**21**	NS4b	1773-1790	QYLAGLSTLPGNPAIASL	DRB1*0404
**22**	NS2	794-810	WPLLLLLLALPQRAYAQ	DRB1*0701
**24**	NS3	1535-1551	TTVRLRAYMNTPGLPVC	DRB1*0701
**25**	NS5b	66-81	VYYLTRDPTTPLARAA	DRB1*0301
**29**	NS5a	1957-1975	SLTITSLLRRHNWITSCS	DRB1*0701

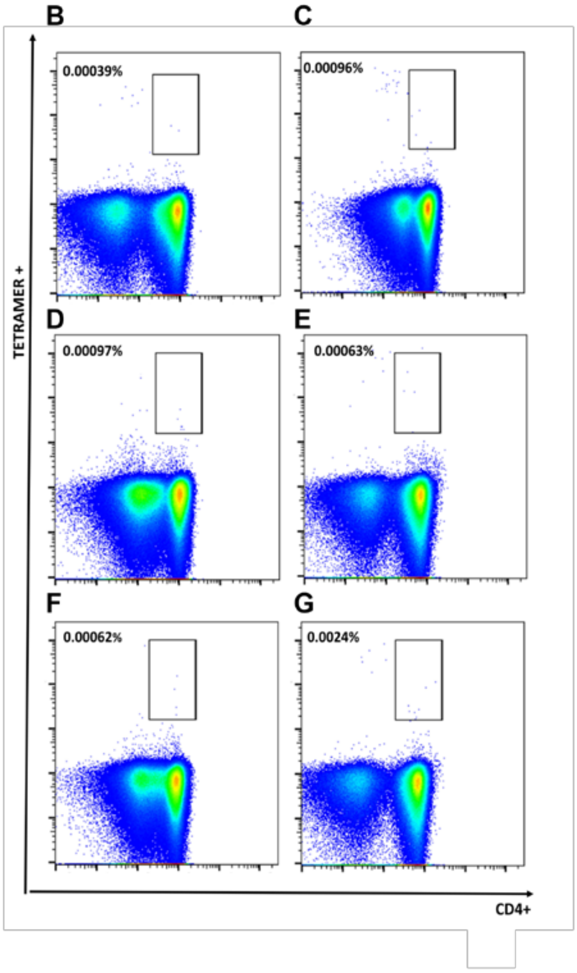

Supplementary Figure 2: **List of MHC class II tetramers**

A) List of MHC class II tetramers encoding immunodominant epitopes IN NS region restricted for specific HLA type. Assigned tetramer number (N), sequence and HLA specificity. B) MHC class II tetramer 14- HLA matched; C) MHC class II tetramer 14- HLA mismatched; D) MHC class II tetramer 17- HLA matched; E) MHC class II tetramer 17- HLA mismatched; F) MHC class II tetramer 24- HLA matched; G) MHC class II tetramer 24- HLA mismatched.

Antibody	Fluorochrome	Concentration	Clone
**Cell surface and intranuclear panel**			
**CD3**	BV570	1:100	UCHT1
**CD4**	AF700	1:100	RPA-T4
**CD127**	PB	1:100	A019D5
**CD28**	PerCP-Cy5.5	1:50	CD28.2
**CCR7**	PE-Cy7	1:50	
**CD45RA**	APC	1:50	HI100
**CD14**	APC-Cy7	1:100	HCD14
**CD19**	APC-Cy7	1:100	HIB19
**Tbet**	PerCP-Cy5.5	1:50	eBio4B10
**Eomes**	PE-Cy7	1:50	WD1928
**Tetramer**	PE	1:100	
**Live dead**	APC-Cy7	1:1000	
**ICS Panel**			
**CD3**	BV570	1:100	UCHT1
**CD4**	BV421	1:100	OKT4
**CD8**	PerCP-Cy5.5	1:100	SK1
**IFN-γ**	AF700	1:50	B27
**TNF-α**	PE-Cy7	1:25	
**IL-2**	APC	1:25	
**MIP-1β**	PE	1:50	
**Live/dead**	APC-Cy7	1:1000	

Supplementary Figure 3: List of antibodies used in FACS experiments

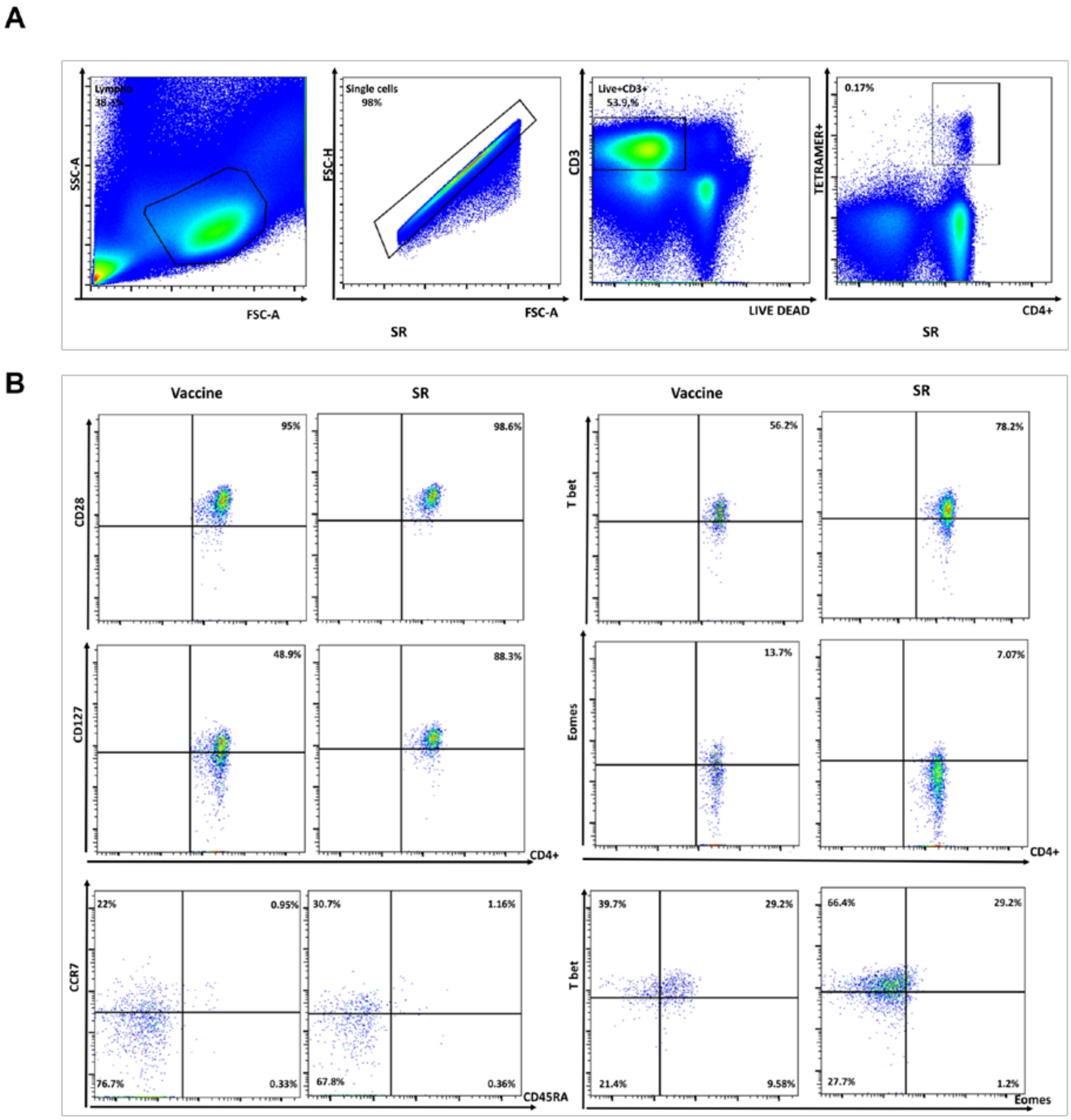

Supplementary Figure 4: FACS plot surface and intranuclear markers on tetramer + CD4+ T cells.

A) Gating strategy used to identify MHC Class II tetramer positive CD4+ T cells.

B) Example gating of cell surface and intranuclear markers used in vaccine (left column)at boost and SR (right column) samples.

A Comparison of tetramer positive CD4+ T cells pre-DAA vs post-DAA therapy

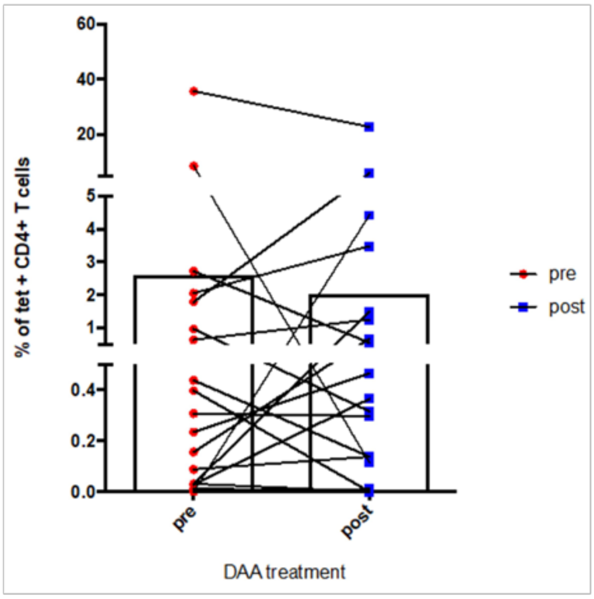

B Subgroup analysis of patients with >2-fold increase of tetramer positive CD4+ T cells vs <2-fold increase following DAA mediated HCV cure

	<2-fold increase	>2-fold increase	P-value
**Age (average)**	58.6y	60.6y	p=NS
**Genotype 1**	9 (70%)	6 (75%)	p=NS
**Genotype 3**	4 (30%)	2 (25%)	p=NS
**Cirrhosis present**	6 (46%)	6 (75%)	p=NS
**Neg RNA <8 wks**	5 (41%)	6 (75%)	p=NS
**Tbet**	0.95	-0.53	p=NS
**Eomes**	-2.3	-1.61	p=NS
**PD-1**	1.1	2.7	p=NS
**Foxp3**	-0.74	0.47	p=NS

C Subgroup analysis of patients with >2-fold decrease of tetramer positive CD4+ T cells vs <2-fold decrease following DAA mediated HCV cure

	<2-fold decrease	>2-fold decrease	P-value
**Age (average)**	59.3y	59.3y	p=NS
**Genotype 1**	11 (73%)	4 (66%)	p=NS
**Genotype 3**	4 (27%)	2 (33%)	p=NS
**Cirrhosis present**	9 (60%)	3 (50%)	p=NS
**Neg RNA <8 wks**	8 (53%)	3 (50%)	p=NS
**Tbet**	-0.45	1.7	p=NS
**Eomes**	-0.7	-3.83	p=NS
**PD-1**	-0.47	-2.92	p=NS
**Foxp3**	-0.16	-0.89	p=NS

Supplementary Figure 5 – DAA subgroup analysis

A) PBMC's cultured with peptide matching tetramer sequence for 14 days. Pre = pre-DAA therapy (red circles), post = post-DAA therapy (blue squares). Mean shown.

B-C) PD-1, Tbet, Eomes and Foxp3 represented as change in expression in post-DAA – pre-DAA for individual samples, with average of individual sample changes represented. Unpaired T-tests used for significance testing.

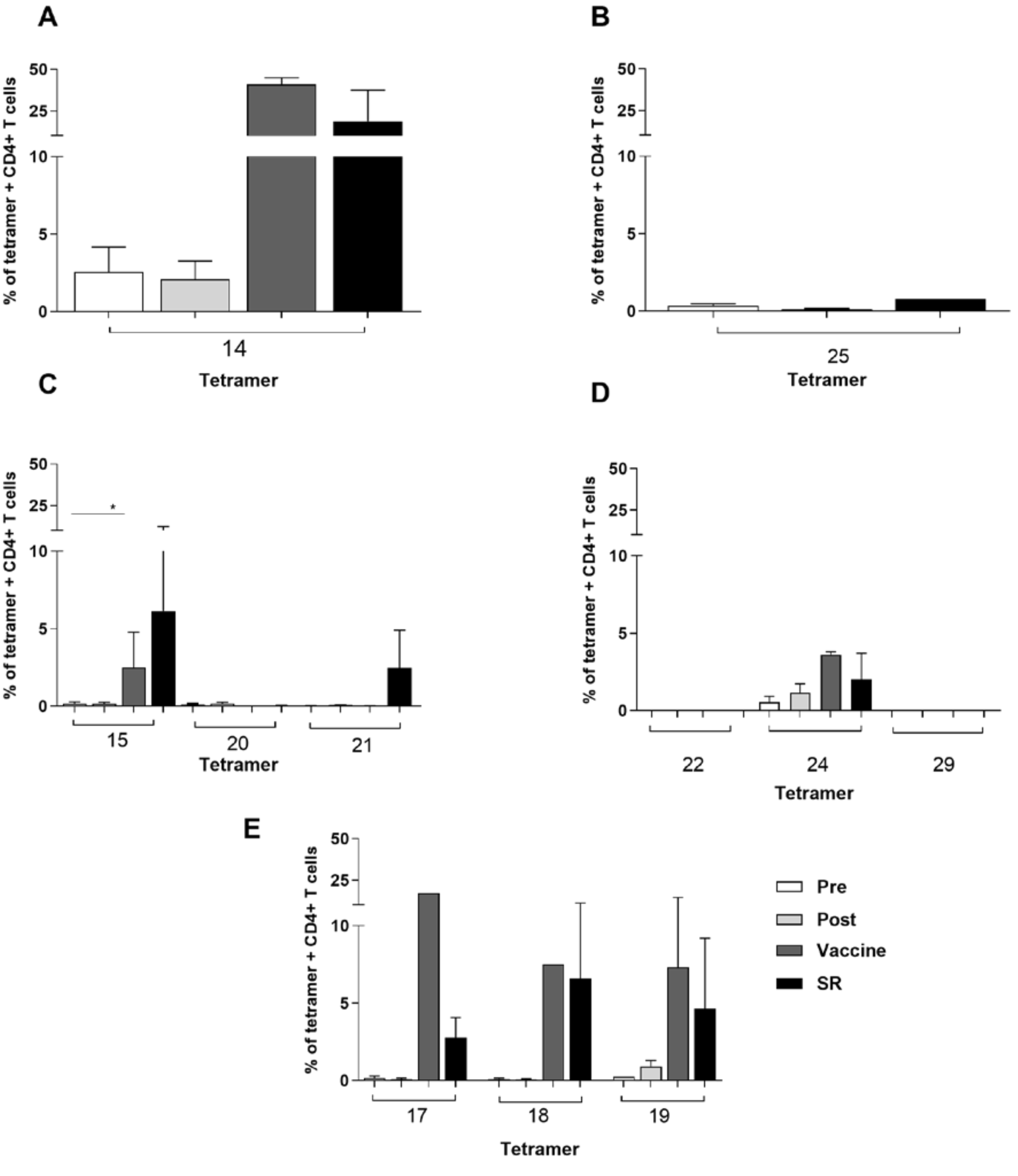

Supplementary Figure 6: Comparison of individual tetramer magnitude following culture.

*PBMC derived from patients undergoing DAA treatment (pre-DAA = white bar, post-DAA = light grey bar), SR individuals (black bar) and vaccinated volunteers (dark grey bar). PBMCs were cultured for 14 days with peptide corresponding to tetramer sequence. Individuals tetramers are represented by HLA Class II restriction. A) DRB1*01 restricted tetramer14 (NS3$_{1806-1818}$), B) DRB1*03 restricted tetramer 25 (NS5b$_{66-81}$), C) DRB1*04 restricted tetramer 15 (NS3$_{1248-1263}$), 20 (NS4b$_{1771-1790}$) and 21 (NS4b$_{1775-1790}$), D) DRB1*07 restricted tetramer 22 (NS2$_{794-810}$), 24 (NS3$_{1535-1551}$) and 29 (NS5a$_{1957-1957}$), E) DRB1*15 restricted tetramer 17 (NS3$_{1582-1597}$), tetramer 18 (NS3$_{1411-1425}$) and tetramer 19 (NS3$_{1535-}$*

1551). Error bars represent the standard error of mean (SEM). Only statistically differences are shown.

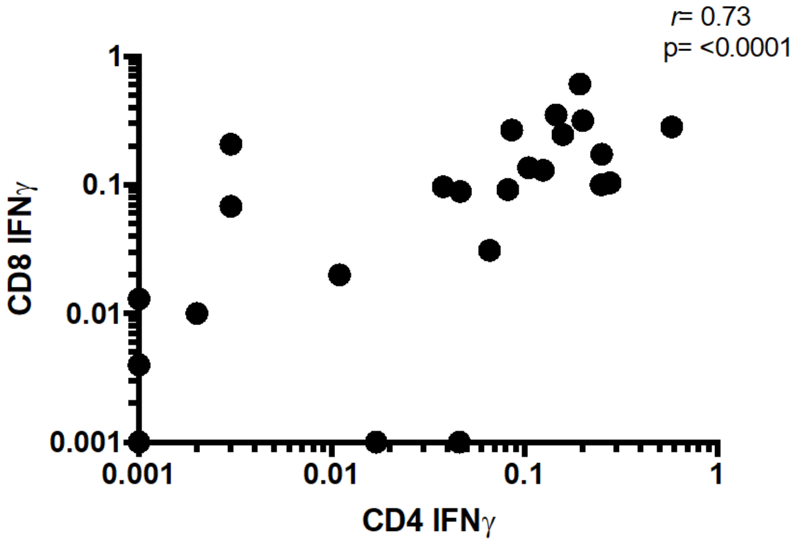

Supplementary Figure 7: Correlation of CD4+ and CD8+ IFNγ production in vaccine induced HCV specific T cells

Frozen PBMCs were cultured overnight with peptide and ICS analysis performed. IFNγ production was measured at different time points following vaccination (following prime vaccination, boost vaccination and end of trial – note values not separated by time). Pearson correlation performed. (results derived from Hartnell et al, Front Immunol 2018)
